# Recent Epidemiology Study Data of Myopia

**DOI:** 10.1155/2020/4395278

**Published:** 2020-11-04

**Authors:** Zhao-Yu Xiang, Hai-Dong Zou

**Affiliations:** ^1^Department of Ophthalmology, National Clinical Research Center for Eye Diseases, Shanghai Key Laboratory of Ocular Fundus Diseases, Shanghai Engineering Center for Visual Science and Photomedicine, Shanghai General Hospital, Shanghai Jiao Tong University, Shanghai, China; ^2^Department of Preventive Ophthalmology, Shanghai Eye Disease Prevention and Treatment Center, Shanghai Eye Hospital, Shanghai, China

## Abstract

Myopia, a pandemic refractive error, is affecting more and more people. The progression of myopia could cause numerously serious complications, even leading to blindness. This review summarizes the epidemiological studies on myopia after 2018 and analyzes the risk factors associated with myopia. The prevalence of myopia varies in different regions, age, and observation time. East Asia has been gripped by an unprecedented rise in myopia, and other parts of the world have also seen an increase. The prevalence of myopia in children continues to rise and aggravates with age. The prevalence of high myopia has also increased along with myopia. Racial dependence and family aggregation can be seen frequently in myopia patients. Increased outdoor activities are proven to be protective factors for myopia, as near-distance work and higher education levels affect in the opposite. The impact of gender or urbanization on myopia is controversial. The relationship between nutrition, digital screens, Kawasaki disease, pregnant women smoking during pregnancy, and myopia is still not clear for lack of sufficient evidence. Understanding the various factors that affect myopia helps to clarify the mechanism of myopia formation and also to formulate reasonable prevention and control measures of myopia to protect people's health, especially for adolescents.

## 1. Introduction

Uncorrected refractive error is not only the second leading cause of global blindness but also the leading cause of preventable visual impairment in children [1]. Myopia, the main manifestation of refractive error, is now an alarming pandemic: 2.5 billion people could be affected by myopia by the end of this decade [2]. In many regions, such as eastern China, myopia is often addressed as a “simple” refractive error, instead of a disease [3]. However, it undeniably increases the risk of diseases of blindness such as macular degeneration, retinal detachment, cataracts, and glaucoma [4–6]. Almost 15 years ago, myopic macular degeneration had already driven myopia to become the leading cause of permanent monocular blindness in Japan [7] and the most frequent cause of severe visual impairment and blindness in the elderly Chinese population in Taiwan [8]. Apart from its deleterious effects on functional vision, the loss of visual acuity associated with uncorrected myopia or permanent vision loss significantly affects all aspects of an individual's quality of life. The constraints that affected individuals experience are likely to further limit their independent choices and pose additional monetary and physical burdens [9]. Furthermore, the economic and financial burden of myopia on families incorporate both the cost of optical devices or other refractive modalities and the need for frequent and long-term management of the condition by an eye-care practitioner [10]. For Chinese urban migrant families, merely the cost of spectacles deters the parents from providing refractive error correction for their children [11], resulting in an increase in myopia and deteriorating functional vision that will certainly damage the future lives of the young. Naidoo et al. reported that the potential global productivity loss associated with the burden of visual impairment was estimated at US$244 billion from uncorrected myopia in 2015 [12]. Controlling myopia, therefore, should be emphasized as a major worldwide public health objective.

## 2. Global Prevalence of Myopia and High Myopia

In 2016, Holden et al. estimated that the global prevalence of myopia was 1.406 billion people worldwide (22.9% of the global population), and that 163 million people had high myopia in 2000. They also concluded that, by 2050, there will be 4.758 billion people with myopia (49.8% of the global population), and 938 million will have high myopia [13]. In accordance with Holden's methodology, we searched PubMed (National Library of Medicine) on March 1, 2020, for epidemiological studies on myopia after January 1, 2018, regardless of the original language of publication. Population-based studies were chosen because they reflect the real-world data of the epidemic. Countries were grouped based on the continent they belonged to. A summary of the data is given in Table 1, showing that the prevalence of myopia varies significantly between different regions, ages, and observation times.

According to epidemiological surveys from the past two years, the prevalence of myopia varies depending on the continent, country, and region. East Asia has been gripped by an unprecedented rise in myopia, and other parts of the world have also seen an increase. As Morgan et al. referred to in their review, the highest rates occur in China, Japan, and Singapore [46]. In China, the highest prevalence occurs in the eastern areas, which are the economically developed parts of China, as shown in Table 1. In South Asia, the prevalence is much lower than in East Asia. In India, the prevalence of myopia is similar to that of the nearby Tibetan province of China where the prevalence is nearly the lowest in all of China. A meta-analysis concluded that only 5.3% of children younger than 16 years of age are myopic in India [47]. The prevalence of myopia in Europe and North America ranges from 6.2% to 26.2% (Table 1).

At present, most of the epidemiological studies of myopia are based on cross-sectional data, while there are relatively few cohort studies. Cohort studies are more informative since they present the annual incidence and progress of myopia, and currently, they all suggest that the prevalence of myopia is increasing every year. According to the published research, the prevalence of myopia among 12- to 17-year-old students in the United States from 1971 to 2004 increased from 12.0% to 31.2%, and over the past 30 years, the prevalence in all ages has increased significantly [48]. A retrospective study of myopia in Taiwan showed that the average prevalence in 7-year-olds increased from 5.8% in 1983 to 21% in 2000; at the age of 12, the prevalence of myopia was 36.7% in 1983 and increased to 61% by 2000 [49]. In southern China, a 5-year follow-up survey was conducted on 6- to 15-year-old children. The cumulative average annual myopia progression was −2.20 D, and the annual change rate of myopia was −0.43 D [50]. Another study in Beijing, North China, showed that the annual incidence of myopia was 7.8%, and the progression of myopia was −0.17 D [51].

A critical parameter for the epidemiological analysis of myopia is age, since prevalence rates have been known to increase significantly with age, as shown in Table 1. In Finland, a total of 240 myopic school children with a mean spherical equivalent (SE) of −1.43 D at baseline were followed up for 22 years, at the end of which, the mean SE of the more myopic eye was −5.29 D. About 32% of the children receiving their first myopic glasses between and around 11 years of age had high myopia (SE ≤ −6.00 D in one eye) in adulthood. A younger onset age of myopia predicted a greater prevalence of high myopia after 22 years, suggested by a prevalence of 65% for those with baseline ages between 8.8 and 9.7 years and 7% for those aged between 11.9 and 12.8 years [52]. An epidemic of high myopia occurs parallel to myopia, as shown in Table 1, perhaps because early-onset myopia progresses more and more before it stabilizes [46].

## 3. The Risk Factors of Myopia

The pathogenesis of myopia is not entirely clear from the current research, and more is believed to be the result of genetic and environmental interactions [53]. The rapid development of the modern economy, the process of industrialization, and the improvement of living standards have all affected the occurrence and development of myopia. Similar to other chronic eye diseases, the risks of myopia can be classified as genetic or environmental factors, the latter of which includes outdoor activities, near-distance work, education, gender, and urban environment, among others, as shown in Table 2.

### 3.1. Genetics/Parental Myopia

The common characteristics of hereditary diseases are race-dependency and familial aggregation, both of which are often seen with myopia. A study based on children of different races found that Asians had the highest prevalence of myopia (18.5%), followed by Hispanics (13.2%), and Caucasians had the lowest prevalence (4.4%) [56]. The apparent familial aggregation of myopia can be shown by the high ratio of parental myopia. A study of Chinese children with an average age of 11.45 years found that the prevalence of myopia in children with one or two myopic parents was 2-3 times higher than that in subjects without parental myopia [53]. In Poland, if both parents are myopic, the odds ratio (OR) of the children having high myopia in adulthood has been shown to be 3.9 [52]. Children with parental myopia also have larger SEs and longer eye axial lengths. To a large extent, family association is considered a genetic factor of myopia, rather than inheritance, because family members have the same environment. However, genetic change cannot explain the rapid changes in prevalence that have taken place over the past one or two generations. Genetics play an important role in early-onset myopia and impose a level of baseline risk, while changes in the environment, especially education and outdoor activities, are the main cause of the emergence of myopia epidemics [46]. To date, more than 25 myopic loci have been discovered via linkage analyses, most of which are on autosomal chromosomes. These loci can be found in the Online Mendelian Inheritance in Man (OMIM) database [57]. A few reports have indicated an interactive effect between genetic predisposition and environmental stress [58]; however, the underlying mechanism remains unclear.

### 3.2. Outdoor Activity

Increasing outdoor activity has been proven to be a protective factor for myopia in many epidemiological investigations, as shown in Table 2. In Guangzhou, 3 years after an increase in outdoor activity in the first grade of a primary school, the accumulation of myopia was 37% lower than that in students without the intervention, and the difference was statistically significant (*P* > 0.05) [59]. Similar results were found in school children in North Ireland, Brazil, and Poland [60–62]. Ho et al. even suggested that 120 min/day of outdoor light exposure during school can prevent the incidence of myopia [63].

The protective mechanism of outdoor activities in relation to myopia is complicated and includes higher illuminance, reduced peripheral defocus, vitamin D, chromatic spectrum of light, physical activity, circadian rhythms, spatial frequency characteristics, and less near-distance work [64]. Among them, higher illuminance is the most well-established theory with evidence shown in both animal and human studies. Norton and Siegwart used animal models to study the relationship between refractive status and light conditions and found that low light (1 to 50 lux) and darkness (<1 lux) are conducive to the extension of the eye axial length, leading to myopia. Strong light (1000–2800 lux), however, delays the occurrence and development of myopia [65]. This effect may be a result of an increase in dopamine receptor D1 activity in the ON pathway [66]. Additionally, Landis et al. measured the amount of time 102 children spent in scotopic (<1–1 lux), mesopic (1–30 lux), indoor photopic (>30–1000 lux), and outdoor photopic (>1000 lux) light during both weekdays and weekends using wearable light sensors, and they found that rod pathways stimulated by dim light exposure are also important in human myopia development. They then suggested that the optimal strategy for preventing myopia with environmental light includes both dim and bright light exposure [67]. Apart from illuminance, many more studies have emerged that focus on the “outdoor light-dopamine” mechanism. Dopamine is a key regulator of both circadian rhythms and eye growth [68]. Natural light from outdoor activities stimulates the retina to secrete more dopamine, and this dopamine was found to control eye growth [69].

We believe that some reported risk factors for myopia may be ascribed to outdoor activity, for example, the seasonal change of myopia growth. Gwiazda et al. found that the speed of myopia progression changes from month to month and is slower from April to September. Therefore, the average progress in winter is higher than that of summer, and the difference is statistically significant (*P* < 0.0001), which may be due to children spending more time outdoors in summer than in winter [70]. In Czech, Rusnak et al. observed 398 eyes of 12-year-old children and found significantly higher axial length growth during the winter period than the summer period. They suggested that the lack of daylight exposure in winter may lead to myopia progression [71].

### 3.3. Near-Distance Work

Many studies have shown that near-distance work is an important risk factor for myopia, such as reading, writing, and working on a computer, as shown in Table 2. Sherwin et al. demonstrated that children working at a distance less than 30 cm had 2.5 times the rate of myopia than those working at longer distances. Additionally, children who would read for more than 30 min at a time had a higher incidence of myopia than children who read for less than 30 min [72]. Research on the effect of near-distance work and eye movement parameters on myopia has speculated that long-term near-distance work maintains the retina image in a defocused state for a long time. Adjusting to the blurred image, then, results in an increased adjustment lag, which, together with other parameters that make chronic hyperopia defocused for a long time, induces the retina to produce some neurotransmitters or growth factors to regulate the inappropriate growth of the eye axial length, leading to the progression of myopia [73]. Working long hours at a close distance and with a low frequency of breaks during study may also be risk factors for myopia, but further research is still needed.

### 3.4. Education

Studies in Singapore, Germany, and other countries found that higher levels of education increase the prevalence of myopia [74, 75]. Previous studies have even shown that the higher the level of education, the higher the prevalence of myopia, as shown in Table 2. Better schools or cram schools have also been shown to be risk factors for myopia [76, 77]. A study that tested the biological interaction of genetic predisposition and the education level on myopia risk found that individuals with high genetic risk combined with a college education have a high risk of myopia, and patients with high genetic risk but only primary education have a much lower risk of myopia [78]. Education may reflect a complex combination of higher levels of exposure to near-reading and correspondingly lower levels of outdoor physical activity, leading to an upregulation of high-risk genes, excessive eye growth, and the development of myopia.

### 3.5. Others

Other myopia-related risk factors such as gender, urbanization, nutrition, digital screens [79, 80], Kawasaki disease [81], and maternal grandmother smoking during pregnancy [82] have been reported, but most of them lack sufficient evidence. Data concerning the effect of gender or urbanization on myopia prevalence, for example, is conflicting. In one study in India on children younger than 16 years old, girls living in urban areas were significantly more likely to have myopia than boys [47], whereas Reed et al. found the opposite to be true [39]. In the same report from Indian, the prevalence of myopia was shown to be higher in urban areas compared to rural areas (OR 2.12) [47], supporting the idea that severe air pollution in cities may accelerate myopia progression [83]. However, Morris et al. did not find strong evidence associating urban or rural status with the incidence of myopia in a United Kingdom cohort of 3,512 children. In that study, the association between the geographical setting and myopia was considered to be potentially driven by underlying confounding factors such as education and time spent outdoors [84].

Nutrition is important for eye development in children and has been suggested to play a role in the incidence of myopia in early life. For example, children who were breastfed during the first 6 months of life were found to be less likely to have myopia [85]. However, the association between diet and myopia is controversial [86, 87]. Recently, there was no significant correlation between an infant's diet at 6, 9, and 12 months and SE, axial length, or myopia at age three years in a Singapore cohort study [88].

## 4. Conclusions

In summary, myopia not only affects the physical and mental health of individuals but also puts a great burden on society. Myopic adolescents are more likely to be anxious than those without myopia [89]. Knowing the various factors that affect the occurrence and development of adolescent myopia is conducive to clarifying the mechanism of myopia formation and also to formulating reasonable prevention and control measures of myopia to protect the health of adolescents.

## Figures and Tables

**Table 1:** Population-based epidemiology study results of myopia and high myopia published from January 1, 2018, to March 1, 2020, in PubMed (National Library of Medicine) database.

Reference	Region, country	Participant number	Age range, year/cohort	Cycloplegia	Mean age (SD), year	Myopia		High myopia	
						Definition	Prevalence (95% CI), %	Definition	Prevalence (95% CI), %
Chen et al. [14]	East Asia, China (East)	43858	Third-year high school students	No	18.4 (0.7) overall	SE < −0.5 D	79.5 (N/A) in 2001 and 87.7 (N/A) in 2015	SE < −6.0 D	8.0 (N/A) in 2001 and 17.5 (N/A) in 2015
Huang et al. [15]	East Asia, China (Taiwan)	6069	6–15	No	10.5 (2.3)	SE < 0.0 D	76.6 (N/A)	SE = −6.0 D	2.2 (N/A)
Wang et al. [16]	East Asia, China (East)	4801	5–20	No	12.3 (3.8)	SE ≤ −0.5 D + UCVA ≤ 20/25	63.1 (61.7–64.5)	SE ≤ −6.0 D + UCVA ≤ 20/25	9.4 (8.6–10.3)
Thorn et al. [17]	East Asia, China (East)	13220	5–16	No	9.4 (1.9)	SE ≤ −1.0 D	49.5 (N/A)	N/A	N/A
Choy et al. [18]	East Asia, China (Hong Kong)	1396	6–13	No	8.8 (N/A)	SE ≤ −0.5 D	37.7 (35.1–40.2)	SE ≤ −6.0 D or AL ≥ 26.5 mm	1.4 (0.8–2.0)
Wang et al. [19]	East Asia, China (southwest)	1626	40–80	No	N/A	SE < −0.5 D	26.4 (24.0–28.7) overall, 31.5 (27.6–35.5) in Han, and 16.8 (12.8–20.8) in Yi	SE < −6.0 D	2.6 (1.8–3.5) overall, 3.3 (2.1–4.6) in Han, and 1.3 (0.2–2.4) in Yi
Wang et al. [20]	East Asia, China (Inner Mongolia)	2090	40–80	No	N/A	SE < −0.5 D	29.4 (27.4–31.3) overall, 31.8 (29.0–34.7) in Han, and 23.0 (19.1–26.9) in Mongolian	SE < −6.0 D	3.6 (2.8–4.4) overall, 4.4 (3.4–5.5) in Han, and 1.5 (0.5–2.5) in Mongolian
Yam et al. [21]	East Asia, China (Hong Kong)	10137 (4257 children and 5880 parents)	6–8 and parents	No^*∗*^	7.6 (1.0) in children and 41.1 (6.0) in parents	SE ≤ −0.5 D (in children) and SE ≤ −0.75 D (in parents)	25.0 (N/A) in children and 72.2 (N/A) in parents	SE ≤ −6.0 D	0.4 (N/A) in children and 13.5 (N/A) in parents
Qian et al. [22]	East Asia, China (Tibet)	3246	Primary and secondary school students	No	12.7 (2.9)	Vision screener score = 0.75	23.8 (N/A)	N/A	N/A
Pan et al. [23]	East Asia, China (rural)	4778 (2432 in grade 1 and 2346 in grade 7)	Grade 1 and grade 7 students	Yes	7.7 (N/A) in grade 1, 13.8 (N/A) in grade 7	SE < −0.5 D	2.4 (1.1–3.7) in grade 1 and 29.4 (28.1–30.8) in grade 7	SE < −6.0 D	0.1 (0.0–0.3) in grade 1 and 0.4 (0.2–0.6) in grade 7
Yotsukura et al. [24]	East Asia, Japan (Tokyo)	1415 (726 primary school students and 752 junior high school students)	6–14	No	10.8 (2.7)	SE ≤ −0.5 D	76.5 (73.4–79.7) in primary school students and 94.9 (93.3–96.5) in junior high school students	SE ≤ −6.0 D	4.0 (2.5–5.4) in primary school students and 11.3 (8.8–13.7) in junior high school students
Ueda et al. [25]	East Asia, Japan (South)	7702 (1892 in 2005, 2874 in 2012, and 2936 in 2017)	≥40	No	Approximately 65 (N/A)	SE < −0.5 D	37.7 (35.5–40.0) in 2005, 40.6 (38.7–42.5) in 2012, and 45.8 (43.9–47.7) in 2017	SE < −5.0 D	5.8 (4.9–6.8) in 2005, 8.0 (7.0–9.0) in 2012, and 9.5 (8.5–10.6) in 2017
Nakamura et al. [26]	East Asia, Japan (rural)	3762	≥40	No	61.8 (14.0)	SE < −0.5 D	29.5 (27.7–31.4)	SE < −5.0 D	1.9 (1.4–2.5)
Lim et al. [27]	East Asia, Korea	3862	5–18	No	11.1 (3.8)	SE ≤ −0.5 D	64.6 (N/A)	SE ≤ −6.0 D	5.4 (N/A)
Singh et al. [28]	South Asia, India (North)	1234	5–15	No^†^	10.5 (3.0)	SE ≤ −0.5 D	21.1 (N/A)	N/A	N/A
Latif et al. [29]	South Asia, Pakistan	1000	10–18	No	13.8 (1.7)	Decided by an ophthalmologist	12.7 (N/A)	N/A	N/A
Hashemi et al. [30]	West Asia, Iran (rural)	2518	16–93	No	44.3 (17.5)	SE < −0.5 D	25.2 (23.2–27.2)	N/A	N/A
Parrey and Elmorsy [31]	West Asia, Saudi	966	16–39	No	27.5 (6.3)	SE ≤ −0.5 D	24.4 (N/A)	SE ≤ −6.0 D	1.7 (N/A)
Yang et al. [32]	Europe, Austria	1507063	18	No	18.0 (N/A)	SE < −0.5 D	13.8 (13.6–13.9) in 1983–1987 and 24.4 (24.2–24.6) in 2013–2017	N/A	N/A
Shapira et al. [33]	Europe, Israel	104689	16–19	No	17.4 (0.6)	SE ≤ −0.5 D	23.3 (N/A) overall, 20.4 (N/A) born between 1971 and 1982, and 26.2 (N/A) born between 1983 and 1994	SE ≤ −6.0 D	1.7 (N/A) overall, 1.5 (N/A) born between 1971 and 1982, and 1.8 (N/A) born between 1983 and 1994
Hagen et al. [34]	Europe, Norway	439 (393 Norwegians)	16–19	Yes	16.7 (0.9)	SE ≤ −0.5 D	13.4 (8.7–18.3) overall and 12.7 (7.9–18.0) in Norwegians	SE ≤ −6.0 D	0.5 (0.1–1.8)
Popović-Beganović et al. [35]	Europe, Bosnia, and Herzegovina	997	7–16	Yes	11.9 (2.6)	SE ≤ −0.5 D	17.3 (15.7–19.0)	N/A	N/A
Harrington et al. [36]	Europe, Ireland	1626 (728 in 6-7 group and 898 in 12-13 group)	6–7 and 12–13	Yes	N/A	SE ≤ −0.5 D	3.7 (2.5–5.4) in 6-7 group and 22.8 (20.1–25.7) in 12-13 group	SE ≤ −5.0 D	0.0 (0.0–0.0) in 6-7 group and 0.4 (0.0–1.2) in 12-13 group
Alvarez-Peregrina et al. 2019 [37]	Europe, Spain	6152 (4159 in 2016 and 1993 in 2017)	5–7	No	6.2 (0.8)	SE < −0.5 D	16.8 (N/A) in 2016 and 19.1 (N/A) in 2017	SE < −6.0 D	1.7 (N/A) in 2016 and 3.6 (N/A) in 2017
Czepita et al. [38]	Europe, Poland	4875	6–16	Yes	11.0 (2.6) in boys and 11.1 (2.6) in girls	SE ≤ −0.5 D	Boys vs. girls: 6–9 (3.7 vs. 3.4), 9–13 (5.7 vs. 8.3), and 13–16 (6.0 vs. 10.4)	N/A	N/A
Reed et al. [39]	North America, U.S.	767	17–38	No^∗^	20.6 (N/A)	SE < −0.5 D	45.0 (N/A)	SE < −6.0 D	1.8 (N/A)
Theophanous et al. [40]	North America, U.S.(California)	60789	5–19	NO^‡^	N/A	SE ≤ −1.0 D	41.9 (N/A)	SE ≤ −6.0 D	2.7 (N/A)
Chiang et al. [41]	North America, U.S.	9960 (6571 for risk factor analysis)	12–19	No	15.4 (0.1)	SE ≤ −1.0 D	31.7 (N/A) in 1999, and 30.2 (N/A) in 2007	N/A	N/A
Mayro et al. 2018 [42]	North America, U.S.(Philadelphia)	18974 2492 (uncorrected refractive errors)	5–12	No^§^	8.1 (1.6)	SE ≤ −0.5 D	9.7 (N/A) overall, and 71.3 (N/A) in patients have uncorrected refractive errors	SE < −6.0 D	0.7 (N/A) in patients have uncorrected refractive errors
Yang et al. [43]	North America, Canada (suburban)	166 (83 in 6–8 group and 83 in 11–13 group)	6–8 and 11–13	Yes	9.6 (2.5) overall, 7.2 (0.6) in 6–8 group, and 12.1 (0.6) in 6–7 group	SE ≤ −0.5 D	17.5 (11.7–23.2) overall, 6.0 (0.9–11.1) in 6–8 group, and 28.9 (19.2–38.7) in 11–13 group	N/A	N/A
Signes-Soler et al. [44]	North America, Mexico	2647	5–14	No^||^	9.1 (1.9)	SE ≤ −0.5 D	4.6 (N/A)	N/A	N/A
Galvis et al. [45]	South America, Colombia	3608 (1933 in 8–17 group and 1675 in 35–55 group)	8–17 and 35–55	No	N/A	SE ≤ −0.5 D + Sph ≤ 0.0 D	12.9 (11.8–14.0) overall, 11.6 (10.2–13.0) in 8–17 group, and 14.4 (12.8–16.1) in 35–55 group	N/A	N/A

SE, spherical equivalent; N/A, not available; UCVA, uncorrected visual acuity. ^∗^Cycloplegic measurements in patients needed a detailed eye examination. ^†^Cycloplegic measurements in 135 patients. ^‡^The last recorded refraction including autorefraction, cycloplegic refraction, and/or subjective refractions. ^§^Cycloplegic measurements in 633 patients. ^||^Cycloplegic measurements in 379 patients.

**Table 2:** Risk factors for the prevalence of myopia.

Risk factors	Reference	Region, country	Odds ratio: prevalence with factor vs. without factor
Parental myopia	Atowa et al. [54]	Africa, Nigeria	6.80 for one myopic parent and 9.47 for two myopic parents
	Yang et al. [43]	North America, Canada (suburban)	2.52
	Harrington et al. [36]	Europe, Ireland	2.4 (paternal)
	Kim et al. [55]	East Asia, Korea	1.84 for myopia and 3.48 for high myopia

Low outdoor activity	Singh et al. [28]	South Asia, India (North)	19.73 (<1.5 hours per day)
	Hagen et al. [34]	Europe, Norway	1.96 (less sport outdoors) and 0.67 (less other outdoors)
	Atowa et al. [54]	Africa, Nigeria	1.25
	Yang et al. [43]	North America, Canada (suburban)	1.17

Time spent on near work/studying/playing	Harrington et al. [36]	Europe, Ireland	3.7 (using screens >3 hours per day) and 2.2 (frequently reading/writing)
	Singh et al. [28]	South Asia, India (North)	2.94 (reading/writing > 4 hours daily) and 8.33 (playing video games > 2 hours daily)
	Wang et al. [16]	East Asia, China (East)	1.88 (moderate school workload) and 2.36 (high school workload)
	Chiang et al. [41]	North America, U.S.	1.27 (watched 2 hours of television daily) and 1.28 (used the computer for 1 hour daily)

High level of education	Wang et al. [19]	East Asia, China (Southwest)	2.50 (undergraduate/graduate)
	Wang et al. [20]	East Asia, China (Inner Mongolia)	1.52 (middle/high school) and 3.77 (undergraduate/graduate)
	Chiang et al. [41]	North America, U.S.	1.79 (senior high school graduate education)
	Yang et al. [32]	Europe, Austria	1.3–1.7 (≥graduated from professional training or served an apprenticeship) in 2013–2017
	Shapira et al. [33]	Europe, Israel	1.16 (≥12 education years)
	Yam et al. [21]	East Asia, China (Hong Kong)	1.12 (mother ≥ undergraduate), 1.10 (father ≥ undergraduate) in children; 1.81 (middle school), 2.78 (high school), 3.47 (associate degree), 5.19 (bachelor degree), and 6.18 (≥master degree) in parents

Female gender	Parrey et al. [31]	East Asia, Saudi	2.56
	Wang et al. [16]	East Asia, China (East)	1.54
	Shapira et al. [33]	Europe, Israel	1.32
	Chen et al. [14]	East Asia, China (East)	1.19 in 2001 and 1.87 in 2015
	Lim et al. [27]	East Asia, Korea	1.15
	Singh et al. [28]	South Asia, India (North)	0.71
	Reed et al. [39]	North America, U.S.	0.61

Urban environment	Latif et al. [29]	South Asia, Pakistan	1.89
	Glavis et al. [45]	South America, Colombia	1.45
	Shapira et al. [33]	Europe, Israel	1.20
	Wang et al. [19]	East Asia, China (Southwest)	1.01
	Wang et al. [20]	East Asia, China (Inner Mongolia)	1.01
High body mass index	Harrington et al. [36]	Europe, Ireland	2.7
Low body mass index	Yang et al. [32]	Europe, Austria	1.2–1.4 in 2013–2017
